# Acceptability and satisfaction of a mindfulness-based healthy eating and stress management program targeting economically marginalized families in a pilot trial

**DOI:** 10.1093/jpepsy/jsaf010

**Published:** 2025-03-17

**Authors:** Sisi Chen, Jiying Ling, Reese Buhlman, Sophia Tadavich, Tsui-Sui Annie Kao

**Affiliations:** Department of Exercise Science, Mercer University College of Health Professions, Macon, GA, United States; Michigan State University College of Nursing, East Lansing, MI, United States; Michigan State University College of Nursing, East Lansing, MI, United States; Michigan State University College of Nursing, East Lansing, MI, United States; Michigan State University College of Nursing, East Lansing, MI, United States

**Keywords:** mindfulness, eating, stress, acceptability, satisfaction, intervention, family

## Abstract

**Objectives:**

To inform and improve future program development, particularly with economically marginalized families, this study aimed to examine the acceptance and satisfaction of a mindfulness-based healthy eating and stress management program among participating parents and daycare teachers in a pilot trial.

**Methods:**

A mixed-methods study was conducted to evaluate a 14-week mindfulness-based program implemented with 107 English-speaking Head Start children (ages 3–5 years) and their parents. The program included a school-based mindful eating curriculum, a home-based parent component to promote mindful eating and reduce parental stress, and a bridging activity connecting home practice with school learning. Quantitative evaluation data were collected from 84 parents (*M*_age_ = 30.12 years) and 12 teachers (*M*_age_ = 43.92 years) via Qualtrics. Semistructured interviews were conducted with 20 parents (*M*_age_ = 31.55 years). Descriptive statistics and thematic analysis were used to analyze data.

**Results:**

Both quantitative (95.2%) and qualitative data demonstrated overall satisfaction with the entire program. About 83%–92% of teachers and 85% of parents considered the school-based curriculum to be satisfactory and acceptable. About 88%–100% of parents were satisfied with the Facebook private group and parent meetings. Approximately 91% of parents found the child letters helpful in connecting and translating school learning into mindful practices at home.

**Conclusions:**

Results demonstrate high levels of acceptance and satisfaction with the mindfulness-based program among economically marginalized families and daycare teachers. Findings provide several key implications for future interventions to incorporate a mindful eating curriculum into daycare routines, proactively connect home practices with school learning to enhance the interactive influence between children and parents, and form a virtual peer support community through social media platforms and group meetings.

Childhood obesity has become a global epidemic and a significant public health challenge (Thomas-Eapen, 2021). In the U.S., obesity affects 19.7% of children and adolescents aged 2–19 years, including 12.7% of preschool children aged 3–5 years (Centers for Disease Control and Prevention, 2024). This is alarming given that obesity is associated with many health risks, including high blood pressure, type 2 diabetes, high cholesterol, breathing difficulties, and joint problems (Centers for Disease Control and Prevention, 2024). In addition, a systematic review of 19 studies found that the median annual obesity incidence was notably higher in preschoolers (4.0%) compared to school-aged children (3.2%) or adolescents (1.8%) (Cheung et al., 2016). This highlights the importance of concentrating intervention efforts on preschool children (Cunningham et al., 2022).

Socioeconomic disparities in childhood obesity are widening worldwide (Ryan et al., 2021). These disparities are not due to individual factors such as poor eating habits and physical inactivity alone but are shaped by systematic factors, including unsafe neighborhoods and limited food access (Jia et al., 2023). Data from the Early Childhood Longitudinal Birth Cohort including 7,022 children aged 4–5 years old indicated that the odds of having overweight or obesity were 70% higher among children from the lowest socioeconomic status (SES) quintile compared to those from the highest quintile (Williams et al., 2018). SES reflects a family’s relative social and economic position, and it is usually determined by factors such as annual household income, parents’ employment status, and education level (Williams et al., 2018).

SES also influences many other key factors contributing to childhood obesity, such as food insecurity and eating behaviors (Gautam et al., 2023; Ortiz-Marrón et al., 2022). Low SES has been associated with unhealthy eating behaviors (Fismen et al., 2021). Children experiencing food insecurity and poor eating habits are more likely to have obesity (Wu et al., 2023; Zhong et al., 2022). For example, one study among 2,022 children aged 2–14 years found that the prevalence of overweight and obesity was more than 10% higher in children with food insecurity compared to those without food insecurity (Ortiz-Marrón et al., 2022).

Given the widening SES disparities in childhood obesity, interventions need to focus on promoting healthy eating behaviors and food security among children from economically marginalized families. However, previous behavior-focused health promotion interventions have shown limited effects on improving behaviors or reducing obesity, particularly among preschool children aged 3–5 years old. For example, one daycare-based program did not yield significant improvements in preschoolers’ healthy eating behaviors or body composition at the 12-month follow-up (Steenbock et al., 2019). Similarly, another internet-based, parent-focused program had no significant effect on reducing preschoolers’ body mass index, although some positive changes in parents’ feeding practices were observed (Hammersley et al., 2019). Notably, these behavior-only interventions were even less effective in children from families with low SES (Coupe et al., 2018; Lockyer & Spiro, 2019).

One potential reason for the limited outcomes, especially in families with low SES, is that these interventions did not consider the high levels of stress parents often experience. Elevated parental stress negatively influences feeding practices, food selection, and preparation behaviors at home. Research shows that higher stress levels are associated with parents’ less healthy feeding practices (González et al., 2022), poorer food choices (Schuler et al., 2020), and reduced availability of healthy foods at home (Jang et al., 2021). Additionally, overwhelming stress can hinder parents’ adherence to obesity prevention programs, weakening intervention effects (Hruska et al., 2020).

To address this important literature gap, we developed the mindfulness-based “*Happy Family, Healthy Kids*” program, a multi-component intervention aimed at improving healthy eating and stress management. The program has shown positive effects on parents’ and preschool children’s healthy eating behaviors, household food security, and anthropometrics (Ling et al., 2024). To effectively guide future program development for young children and families with low SES, it is essential to qualitatively understand participants’ experience, acceptance, and satisfaction. However, qualitative appraisal of intervention programs is often limited. To offer important insights for future improvements, this mixed-methods study examined the acceptance and satisfaction of the “*Happy Family, Healthy Kids*” program among participating parents and daycare teachers in a pilot trial.

## Methods

### Study design

This mixed-methods study was part of a pilot, one-group, nonrandomized quasiexperimental clinical trial conducted in 2021–2022. The pilot trial’s primary aims were to examine the preliminary effects of the program on improving eating behavior, food insecurity, and anthropometrics among 107 children (aged 3–5 years old) and their parents recruited from 23 Head Start daycare classrooms in the Midwestern U.S. (Ling et al., 2024). Due to the global pandemic, the originally proposed in-person parent group meetings were transitioned to a virtual format. No additional modifications to the methods were made after the trial commenced. Children aged 3–5 years old, and their primary caregivers (adult parents or legal guardians) were eligible for the trial if they could speak and understand English. The goal of this convergent mixed-methods study was to appraise parents’ and daycare teachers’ experiences (i.e., acceptance and satisfaction) with the program. Semistructured qualitative interviews (1-on-1) were performed to understand parents’ and teachers’ experiences with various aspects of the program. Acceptance and satisfaction were also quantitatively evaluated by the participating parents and teachers. The pilot clinical trial was approved by a University Biomedical and Health Institutional Review Board. Parental consent and child verbal assent (if ≥5 years old) were obtained before data collection. Informal consent was also obtained from teachers who participated in data collection. We followed the CONSORT 2010 checklist for reporting, and the finalized checklist is provided as Supplementary File S1.

### Intervention

Details of the intervention description can be found in a prior manuscript (Ling et al., 2024) and are further summarized in Table 1. The 14-week mindfulness-based “*Happy Family, Healthy Kids*” program was grounded in the Actor–Partner Interdependence Model (Cook & Kenny, 2005), Social Cognitive Theory (Bandura, 2001), Allostatic Load Model (McEwen, 2000), and Transactional Theory of Stress and Coping (Lazarus & Folkman, 1987). It utilized mindfulness practices to mitigate obesogenic behaviors/risks for Head Start preschoolers and their parents. The program contains 3 components: (a) a school-based mindful eating curriculum called “Eat my ABCs” taught by trained daycare teachers or external interveners to increase children’s healthy eating knowledge and mindful eating skills. This curriculum follows an alphabet theme and incorporates school readiness content such as recognizing letters, numbers, shapes, colors, sizes, and basic counting skills; (b) a home-based healthy eating and stress management component (e.g., Facebook private group, 3 virtual parent group meetings with guided yoga practice) to promote mindful eating and reduce parental stress; and (c) a bridging activity (letters made by children using stickers) connecting home practice with school learning to facilitate mindful eating behavior at home. Each family received a program cookbook and MyPlate plates to assist positive eating behavior changes at home. Parents were asked to complete 4 weekly tasks and quizzes via a private Facebook group and/or the study website. The discussions through Facebook were facilitated by research assistants to provide educational materials and simulate positive interactions among parents. Three parent meetings via Zoom were implemented to improve parents’ abilities to handle picky eaters, cope with stress, eat healthy with MyPlate, read food labels, and utilize community resources to stay healthy. Each parent meeting (lasting about 60 min) was offered at 2 time slots: Wednesday 6–7 PM and Friday 10–11 AM.

**Table 1.** “Happy Family, Healthy Kids” intervention.

Component	Description
School-based “Eat My ABCs” mindful eating curriculum	14 weeks, 20 min/lesson/week, taught by trained childcare teachers or external interveners.
	Each lesson consisted of 2 components, focusing on learning 2 fruits/vegetables using 5 senses: one on fruit/vegetable learning, and the other on mindful eating food-tasting activity.
	The last lesson was a review session focusing on the 26 fruits/vegetables learned.
Virtual parent program	14 weeks with 4 weekly tasks: *Raise a Happy Family, Get in the Kitchen, Leave a Positive Comment*, and *Take a Quiz*.
	Private Facebook group hosted weekly program videos and flyers on healthy eating and stress management strategies. Parents posted weekly about healthy eating and stress management.
	Private study website hosted all study materials and weekly quizzes.
	Three virtual parent meetings via Zoom with parents (children could attend as well) at weeks 1, 7, and 14 focusing on yoga practices, healthy behavioral changes, and stress management strategies at home.
	Three weekly motivational text messages (Monday, Wednesday, and Friday) to encourage parents in supporting healthy behavioral changes and stress management at home.
Bridging activity connecting home practice with school learning	13 letters for the first 13 lessons of the child mindful eating curriculum.
	After each child lesson, children created a letter using colorful stickers to share with parents what they had learned in school and what they wanted to try at home. The completed letter was sent to each parent privately via text or Facebook Messager each week.

### Quantitative data collection and measures

All quantitative evaluation data were collected via Qualtrics. At the end of each parent meeting, attended parents were asked to complete a meeting evaluation survey. After the intervention, appropriate online survey links were shared with consented teachers and parents through text messaging and/or emails. No incentive was provided to teachers for completing the teacher evaluation survey or to parents for completing parent meeting evaluations. A $10 e-gift card was provided to each parent who completed the postintervention survey containing program evaluation questions. Supplementary File S2 contains all quantitative evaluation surveys and the parent interview guide.

#### Teacher evaluation

Teacher evaluation of the child curriculum’s acceptability and satisfaction was assessed by a 14-item investigator-developed survey that contained 11 close-ended and 3 open-ended questions. Each close-ended question had 4 response choices ranging from strongly disagree to strongly agree. Responses of “agree” and “strongly agree” were combined to indicate that the curriculum was acceptable or satisfactory. One example question is, “Overall, I am satisfied with the curriculum.” After the program ended, 18 daycare primary teachers from 23 classes (5 teachers participated in both year 1 and 2) were contacted to complete the evaluation survey via Qualtrics.

#### Parent meeting evaluation

Parent meeting evaluation was conducted after each parent meeting. Each meeting evaluation included 3 close-ended and 2 open-ended questions assessing parents’ satisfaction with the guided yoga practice, meeting content relevance, recommendation to other parents, perceived barriers to attending, and suggestions for additional content. Responses of “somewhat satisfied/helpful” and “very satisfied/helpful” were interpreted that the meeting was acceptable or satisfactory.

#### Parent evaluation

Parent evaluation of the entire program’s acceptability and satisfaction was assessed by a 15-item survey. The questions, developed by the research team, focused on obtaining feedback on the overall program satisfaction and acceptance/usefulness of intervention components, including the cookbook, Facebook-based activities, and child letters. One example question is, “How helpful were the weekly letters made by your Head Start child?” Responses of “agree a little,” “somewhat helpful/satisfied,” “agree a lot,” or “very helpful/satisfied” were combined to indicate that the program was considered acceptable or satisfactory by parents.

### Qualitative data collection

Following the previously pilot-tested interview guide (Zahry & Ling, 2020), trained interviewers, such as research assistants who were not directly involved in delivering the intervention to parents, conducted semistructured interviews with each consented parent via telephone calls and audio recorded all interviews. Parents provided consent for participation in interviews during the enrollment survey process. Consented parents from each daycare center were then randomly selected and invited to participate in the interviews until data saturation was achieved to ensure a representative sample. The interview guide contained questions on evaluating the overall program, parent meetings, the private Facebook group, the study website, and the child letters sent to parents. The same set of interview questions was asked in every interview. The interviews averaged 22 min in duration. All audio recordings were transcribed by the LANDMARK Associates Inc. and then manually checked and verified by 2 research assistants independently with the original audio recordings. The sample size of qualitative interviews was determined by achieving data saturation in which no new information could be obtained from additional interviews.

### Data analysis

Quantitative data were analyzed using descriptive statistics, including means, standard deviations, ranges, frequencies, and percentages, with SPSS Statistics version 28. Thematic analysis (Kiger & Varpio, 2020) was used to analyze qualitative data depicting parents’ experiences with various intervention components. J.L. and T.-S.A.K. developed the coding scheme based on a deductive approach (Azungah, 2018), drawing from the research and interview questions. R.B. and S.T. then independently coded each interview transcript following this deductive framework. During the coding process, inductive codes also emerged, reflecting new themes or insights not anticipated in the initial coding scheme. R.B. and S.T. met to discuss their coding results, resolving discrepancies until reaching a consensus. Agreed codes were then grouped into preliminary themes, capturing patterns and central ideas relevant to each intervention component. J.L. further reviewed and refined these themes to ensure they accurately reflected the original data. Finally, the resulting themes were then integrated with quantitative findings through data triangulation to provide a comprehensive understanding of participants’ experiences (Fielding, 2012), including perceived acceptance and satisfaction with each intervention component.

## Results

### Participants

The pilot clinical trial was conducted among 107 children and their parents (see Table 2). Out of 107 parents, 91 (85.1%) provided consent for an individual interview. Twenty-six parents were contacted, and 20 (76.9%) completed the qualitative interviews. Six parents did not complete the interviews, primarily due to nonresponse after a week or cancellations caused by family conflicts. No harms or unintended effects were reported throughout the study. The mean age of these 20 parents was 31.55 years old (*SD * = * *4.76), 1 (5%) was Hispanic, 3 (15%) were Black, and 9 (47.4%) were single. Eight (42.1%) had annual family income <$20,000, 8 were unemployed, and 8 had an education level of high school or lower. On average, each interviewed parent had about 2 children living in the household. As shown in Table 2, there was no significant difference in demographics between the 107 parents and 20 interviewed parents.

**Table 2.** Demographics of study parents.

Variable	Study sample (*n* = 107)		Interview sample (*n* = 20)		Comparison	
	*M* or *n*	*SD* or *%*	*M* or *n*	*SD* or *%*	*t* or *χ^2^*	*p*-value
**Age** (years)	30.12	5.98	31.55	4.76	1.19	.119
**Sex** (female)	102	95.3%	20	100%	1.21	.581
**Ethnicity** (Hispanic)	7	6.5%	1	5%	.10	1.00
**Race**					2.83	.610
White	79	73.8%	14	70%		
Black	16	15%	3	15%		
Multiracial	8	7.5%	3	15%		
Other (Asian, Native)	4	3.7%	0	0		
**Marital status**					2.52	.310
Married or partnered	51	51.5%	10	52.6%		
Separated, divorced, or widowed	9	9.1%	0	0		
Single	39	39.4%	9	47.4%		
**Annual family income**					3.53	.328
<$20,000	44	44.4%	8	42.1%		
$20,000–29,999	23	23.2%	2	10.5%		
$30,000–49,999	23	23.2%	6	31.6%		
≤ $50,000	9	9.1%	3	15.8%		
**Employment status**					.39	.894
Full-time	35	35.4%	6	31.6%		
Part-time	21	21.2%	5	26.3%		
Unemployed	43	43.4%	8	42.1%		
**Education level**					3.37	.656
<High school graduate	11	11.1%	1	5.3%		
High school graduate	38	38.4%	7	36.8%		
Some college	32	32.3%	6	31.6%		
Technical school or community college degree	11	11.1%	2	10.5%		
≥ Bachelor’s degree	7	7.1%	3	15.8%		
**Number of children living in the household**	2.80	1.36	3	1.53	.72	.238

Twelve (66.7%) out of 18 daycare teachers completed the quantitative evaluation survey on the school-based mindful eating curriculum (see Table 3). The mean age of participating teachers was 43.92 years old (*SD * = * *12.34, range: 25–61) with one male teacher. Eleven (91.7%) were white, 10 (83.3%) were married or partnered, 4 (33.3%) had an annual family income between $30,000 and $49,999, and 10 (83.3%) had a bachelor’s degree. On average, teachers had worked 9 years in Head Start (*SD * = * *5.44, range: 3–21).

**Table 3.** Demographics of participating daycare teachers (*n* = 12).

Variable	*M* or *n*	*SD* or *%*
**Age** (years, range: 25–61)	43.92	12.34
**Sex** (female)	11	91.7%
**Ethnicity** (non-Hispanic)	12	100%
**Race**		
White	11	91.7%
Other	1	8.3%
**Marital status**		
Married or partnered	10	83.3%
Separated, divorced, or widowed	2	16.7%
**Annual family income**		
$30,000–49,999	4	33.3%
≤ $50,000	7	58.4%
Missing	1	8.3%
**Education level**		
Technical school or community college degree	1	8.3%
Bachelor’s degree	10	83.3%
Graduate or professional degree	1	8.3%
**Number of years worked at Head Start (range: 3–21)**	9.00	5.44
<5 years	2	16.7%
5–10 years	8	66.6%
>10 years	2	16.7%

### Evaluation of intervention components

#### School-based mindful eating curriculum

##### Positive reception and satisfaction

All 12 daycare teachers who completed the quantitative evaluation survey endorsed the curriculum consent as being informative. Eleven (91.7%) stated the curriculum was age-appropriate for preschoolers and the curriculum instructions were easy to understand. Ten (83%) agreed that the curriculum met their expectations and increased children’s knowledge and interest in trying fruits/vegetables. Eleven (92%) showed high levels of confidence in teaching the curriculum themselves. Taken together, 83% of teachers were satisfied with the child curriculum. One teacher [25 years old, White, with 5 years of experience as a childcare teacher] who was not satisfied with the curriculum suggested focusing on one fruit or vegetable per week instead of 2.

##### High demand and potential sustainability

The additional feedback obtained from teachers via open-ended questions further advocated the significance of our program. For example, teachers shared, “*I think there is a true need for such programs because the Head Start audience often lacks knowledge of and access to fresh fruits and vegetables and different kinds of fruits and vegetables*. [48 years old, White]” and, “*The need for the programs is huge. We need to encourage parents to make healthy decisions when preparing meals and the opportunities/incentives your team has given my families is great. I would love to see this program continue for our families*. [47 years old, White]” Additionally, teachers endorsed the easiness of implementing the curriculum in their classrooms by saying, “*The way the curriculum was set up worked great for me. I had everything I needed in a convenient folder, separated, organized, stickers in baggies. I found this to be extremely helpful!* [with 21 years of experience as a childcare teacher]” As a result, 9 (75.0%) stated that they would incorporate the curriculum in their future teaching. One teacher [25 years old, White], who would not incorporate the curriculum into her future teaching, shared that the primary barriers families had in supporting balanced eating habits at home were limited access to food and low SES.

##### Positive impact on healthy eating habits

Eleven teachers (92%) had observed their classroom children eating more fruits/vegetables after participating in the program. Participating parents were also impressed by this school-based child curriculum. Among 20 parent interviewees, 17 (85.0%) described the child curriculum to be “*beneficial*” and “*intriguing*.” For example, one parent [58 years old, Black] shared: “*It influenced me to cook healthier foods. It also informed my child to eat healthier.*” Another parent [36 years old, White] stated: *“She likes [the program]. She can’t communicate but yet since she did this program at school, she started learning more ways of communicating with us about her food and let us know this is yummies or yuck.*” Many parents observed positive changes in their children’s eating behavior at home. More specifically, after receiving the child lessons at daycares, one parent [54 years old, White] discovered her child “*loves peppers*” so she “*put peppers in little baggies like they are chips or crackers*” to make healthy lifestyle changes at home, and another parent [41 years old, Black] found her child was “*actually eating vegetables such as carrots and broccoli*.” Parents attributed their children’s changes in eating behaviors (↑fruits/vegetables) at home to the school-based mindful eating curriculum.

#### Home-based parent component

A postintervention quantitative evaluation survey (*n*  =  84) and individual qualitative interviews (*n*  =  20) were conducted to obtain an in-depth understanding of parents’ experiences with the home-based component. In addition, 58, 45, and 39 parents attended meetings 1, 2, and 3, respectively, with quantitative evaluations completed by 40, 36, and 28 parents for each meeting.

##### Acceptability and positive impact of the private Facebook group

Among the 84 parents who completed the postintervention quantitative evaluation survey, 76 (90.5%) stated the materials posted in the Facebook private group were easy to understand, and 74 (88.1%) said the materials assisted them in helping their families to have a healthy lifestyle. From qualitative interviews, 17 (85%) displayed a positive experience regarding the Facebook interactions. Some parents stated that the Facebook private group fostered a virtual learning community for them to learn from each other. By observing other parents’ posts, parents were motivated to “*be more involved*” and “*be able to expand [their] horizon*” with different healthy lifestyle ideas, activities, and recipes to try with their children. One parent [45 years old, multiracial] stated: “*I saw a lady who had said that she included her kids on her walking and exercising. That made me feel, ‘Well, if she has kids and can do this, then I can do it too.’*” Another parent [42 years old, White] shared that when she was encountering “*a very difficult time*,” she “*took a couple of the stress management tips that I thought would work*,” which helped her to overcome undue stress and anxiety. According to the quantitative data, 75 parents (89.3%) responded that they would use the intervention information in the future, and 74 (88.1%) would recommend the program to other parents. Reasons for not recommending included not using or not enjoying using Facebook or feeling uncomfortable connecting with other parents through the Facebook group.

##### Doable tasks and quizzes

From qualitative interviews, participating parents shared their overall experience with the weekly tasks and quizzes delivered through the private Facebook group and/or the study website. Seventeen (85.0%) interviewed parents appreciated the weekly tasks and quizzes. They described the tasks and quizzes as “*engaging*,” “*easy to complete*,” and made them “*look at things differently*.” For example, one parent [42 years old, White] described: “*I definitely thought that the weekly tasks were for sure doable, and then the quizzes were short, sweet, simple.*” Another parent [36 years old, White] shared: “*The weekly task brings where everybody can see what someone else is doing for a stress reliever. Some parents were doing their own dinners and sharing those ideas, so it gave us all something different to look at.*” Twelve (80.0%) parents agreed that the frequency and number of tasks and quizzes per week were very acceptable, even when they were always “*running, running, running*” around their work schedule and childcare activities. They also shared that the weekly reminders for completing tasks and quizzes were very helpful in keeping them on track.

##### Challenges to participation in the Facebook discussions

In the postintervention quantitative evaluation survey, parents shared the barriers that stopped them from participating in the Facebook discussions were their busy schedule, mainly due to excessive work, multiple kids, or being a single parent; illnesses among family members; unreliable internet; and mental health issues such as social anxiety. The qualitative interviews echoed some identified barriers, including feeling “*distracted*” and “*overwhelmed*” from their workload and care of their kids, limited use of social media such as Facebook, and no reliable access to the internet.

##### Interactive and influential parent meeting

Based on the quantitative evaluation surveys, all parents found the 3 offered meetings’ contents helpful, and 99.0% (*n * = * *103) would recommend these meetings to other parents. During the qualitative interviews, parents described the parent meetings as “*interactive*,” “*valuable*,” and “*informative*,” though some noted they could be “*challenging in navigating*” due to the 1-hr duration, time constraints, and competing family responsibilities. More specifically, parents shared that the meetings allowed them to learn how to shop smartly to save money, read food labels to check for nutrition facts, and apply deep breathing to delay undesired reactions (anger) to an unpleasant event. For example, one parent [38 years old, White] shared: “*My favorite one was when we learned about the food groups like reading the labels.*” Another parent [58 years, Black] stated: “*I think that one was my favorite of the three, because it was more about stress and coping. I felt really good talking about that, and I liked that we started with that.*” All interviewed parents felt more engaged with the program upon the completion of the parent meetings.

##### Favorability of yoga practices: enhancing bonding and relaxation

The parent meeting quantitative evaluations revealed that 95% (*n * = * *38), 94.4% (*n * = * *34), and 100% (*n * = * *28) of parents were satisfied with the beginning yoga session at each parent meeting, respectively. This theme was also reflected in the qualitative data in which all interviewed parents consistently identified the guided yoga practice as their favorite activity throughout the parent meetings. They expressed that the yoga practice made them feel “*peaceful*” and in “*a Zen mood*.” One parent [58 years, Black] shared: “*I definitely loved it. Yoga is great. It’s relaxing, and it kind of takes the tension away from you, making you more relaxed, so then you can enjoy the meeting better.*” Additionally, parents stated that the yoga session provided an important bonding opportunity with their children. Some parents even stated that doing yoga together was fun and had become a family routine for their families. The comments “*my daughter likes doing it with me*,” and “*my kids do it with me every now and again*” consistently appeared in the qualitative interview data.

##### Barriers to not attending meetings

The largest barrier to not attending meetings was the lack of time due to a busy working schedule and completing family responsibilities (e.g., caring for sick children, transporting children to and from school). This barrier consistently emerged in both quantitative (*n * = * *43, 41.3%) and qualitative data. Also, the distraction from children (*n * = * *9, 8.7%) also prevented them from being fully engaged in meeting activities, based on the quantitative data.

#### Child letters connecting home practice with school learning

##### Child letters enhanced parent home eating practices

To ensure school learning is well-informed and translated to parents at home, weekly child letters (made by children at daycares) were implemented and evaluated. Participating families had noticed significant changes in their positive eating behavior after beginning to receive these weekly child letters. From the quantitative postintervention evaluation survey, 76 (90.5%) parents found the child letters helpful, and 71 (84.5%) had discussed the content of letters with their children. Additionally, 73 (86.9%) had followed the content of letters accordingly and further shopped and prepared nutritionally balanced foods for their children at home. The main reasons for not benefiting from the child letters were either not receiving the child letters from daycare teachers (*n * = * *4, 4.8%) or children frequently missing lessons due to illness (*n * = * *3, 3.6%).

In the qualitative data, parents shared that their child was “*proud*” and “*got so excited*” with every letter they brought home. They even reminded their parents to go to the grocery store and buy the foods they had tried at school. For instance, one parent [54 years old, White] shared: “*Me and my daughter loved those. In fact, she would remind me every time I went to the grocery store. Like the dragon fruit, she’d say, ‘Get my dragon fruit, dragon fruit, please.’ She insisted on reminding me what fruits and vegetables to get for her. I absolutely loved those. I thought they were the cutest ever [laughter].*”

##### Child letters propelled the entire family to eat healthier

Parents also noted that the discussions and actions around child letters had propelled the entire family to eat healthier at home. Parents stated that they were able to “*expand their horizon*” by trying and tasting the foods described in the letters. One parent [40 years old, White] shared her experience, stating: “*I enjoyed it. There was Jicama I’ve never heard of, and we tried it actually in the air fryer and made fries out of it. I actually enjoyed it. I learned about different fruits and vegetables I’ve never tried a day in my life. Normally, I would’ve said, ‘Uh, no, I don’t eat fruits and vegetables. I’m not trying that.’ But my son was like, ‘Come on, just try it.’*”

##### Developmental barriers to communication in young children

Despite the positive comments above, 3 interviewed parents (15.0%) commented that they were “*having difficulty communicating*” with their young children about the school activities due to their young age or delayed development and subsequently could not follow the content described in the child letters. These parents reported limited benefits from receiving child letters mainly because their children were either too young to “*really talk (yet)*” or were not able to share a lot due to developmental delays.

##### Overall program evaluation

Results derived from both quantitative (95.2%) and qualitative data demonstrated an overall satisfaction with the entire program. According to the quantitative data, 74 (88.1%) parents expressed that they would participate again if given chances. A general satisfactory perception can be concluded by the following quotes “*The program was amazing! Always adjusted to my schedule when needed*;” and “*My kids loved the program*!” In addition, some teachers stressed the important impact of the program to the families they served and stated: “*This goes hand to hand with our program and its nature for families to strive.*”

## Discussion

This study investigated the acceptance and satisfaction of the mindfulness-based, healthy eating, and stress management program, “*Happy Family, Healthy Kids*,” among participating parents and daycare teachers in a pilot trial. The results revealed 3 key findings: (a) overall acceptance and satisfaction with the program; (b) the program effectively addressed unhealthy eating habits and high levels of stress experienced by both children and parents; and (c) practical elements that support the program’s feasibility for implementation by teachers and parents at both school and home settings. These findings have implications for enhancing our implementations of mindfulness-based, healthy eating, and stress management programs to curb the current childhood obesity epidemic, particularly for families with low SES.

The present study builds upon and extends prior literature (Ling et al., 2024) by demonstrating that both parents and daycare teachers expressed strong acceptance and satisfaction with the school-based mindful eating curriculum. This may have occurred because the curriculum incorporated school readiness content, addressing a critical barrier noted in previous research. Carraway-Stage et al. (2014) identified meeting school readiness priorities as a primary challenge to implementing food-based learning in daycare settings. By aligning with these educational priorities, the current intervention successfully overcame this challenge, enabling daycares to fulfill their core missions while simultaneously promoting lifelong healthy eating habits (Dixon et al., 2023). This dual-purpose approach adds a unique contribution to the literature by illustrating how mindful eating curricula can align with institutional goals, making them more feasible and appealing for implementation. Moreover, mindful eating education can not only improve children’s eating behaviors but also benefit their social and emotional development through enhancing self-regulation and awareness (Schmitt et al., 2020). This evidence emphasizes the need for policy initiatives that integrate mindful healthy eating curricula into early childhood education standards. Such policies could play a pivotal role in addressing the ongoing childhood obesity epidemic and fostering a healthier, more resilient generation in the U.S.

Results from this study support the feasibility and success of using social media platforms to engage parents in health promotion interventions, aligning with previous literature emphasizing the effectiveness of social media peer groups in reaching families with low SES whose children are at high risk for obesity (Fiks et al., 2017). Social media offers unique opportunities for peer interaction and support, fostering a sense of connectedness among parents, which can help alleviate parental stress and encourage the adoption of healthier family behaviors (Berge et al., 2020; Fiks et al., 2017). The convenience and accessibility of social media make it particularly suited to engaging hard-to-reach populations (Phillips & Spratling, 2024), addressing common barriers such as time constraints and geographic limitations. However, ethical considerations such as privacy protection and data security must be rigorously addressed when utilizing social media in research (Bhatia-Lin et al., 2019).

The virtual parent meeting format helps overcome the major barrier of time constraints often faced by parents with young children (Hesketh et al., 2017), creating opportunities for families to implement healthy behaviors in their daily lives. However, meeting days and times need to be carefully planned to accommodate these families’ irregular work schedules, often influenced by multiple shifts, restaurant jobs, or retail store hours (Zahry & Ling, 2020). Parents particularly appreciated the inclusion of guided yoga practices in each meeting because it provided a fun and valuable bonding opportunity with their children, helped regulate their moods and emotions, and improved their mental health (Pascoe et al., 2021). The serene connection cultivated through practicing yoga between children and parents can nurture a positive relationship, potentially lowering the risks of childhood obesity (Niec et al., 2022). These findings underscore the importance of incorporating mindful activities (e.g., yoga) into virtual parent meetings, as they provide a dual benefit of enhancing mental health and supporting obesity prevention efforts.

The simple child letters served as an essential bridge between school learning and home practices, leveraging intergenerational influence to improve children’s health in their natural environments as they grow. Young children are more likely to try foods they are familiar with (Amsel et al., 2019) and those they have helped prepare (DeJesus et al., 2019). In this study, children sampled a wide range of fruits and vegetables during daycare activities and then conveyed their preferences to parents through personalized child letters. These letters encouraged parents to actively involve their children in food preparation at home, strengthening the connection between classroom learning and household habits. Such innovative strategies likely contributed to the observed improvements in healthy eating behaviors among both children and parents (Ling et al., 2024). Given the significant role of parents in shaping children’s healthy lifestyles (Coto et al., 2019) and the common developmental delays among preschoolers from low-SES backgrounds (Han et al., 2024), this sustainable practice of using child letters with colorful stickers has the potential to positively influence the behaviors and overall health of entire families. By fostering active parent–child engagement and reinforcing healthy habits at home, the approach strengthens the connection between early education and family practices, amplifying the program’s long-term impact.

### Limitations

This study has several limitations. First, children’s perspectives on the intervention were not directly assessed due to their young ages (3–5 years old). Thus, their views on the acceptability and satisfaction with the program remain unknown. Furthermore, standardized measures of acceptability and satisfaction, such as the Client Satisfaction Questionnaire (Attkisson & Zwick, 1982), were not utilized, which could limit comparability with other studies. Second, the study’s generalizability is constrained by its focus on economically marginalized families with internet access. However, the Pew Research Center (2024) reported that 97%–98% of the U.S. adults aged 18–49 years had internet access in 2023. Also, the ethnic and racial distribution of the study participants (5% Hispanic, 15% Black) closely aligns with state census data (6% Hispanic, 14% Black) (United States Census Bureau, 2023), indicating reasonable representativeness in these demographic aspects. Additionally, only 20 out of 107 participating parents completed individual interviews, which may introduce selection bias, although no significant demographic differences were noted between interview participants and nonparticipants. Lastly, the study occurred during a global pandemic context, potentially introducing confounding factors such as heightened stress or altered routines, which could have influenced participant behaviors and outcomes.

## Conclusions

Results from this mixed-methods pilot study indicate the potential acceptance and satisfaction with the mindfulness-based program (emphasizing both healthy eating and stress management) among economically marginalized families and daycare teachers. The findings suggest several implications for future interventions to prevent childhood obesity. Incorporating mindful eating curriculum into daycare school readiness routines may be feasible and acceptable to both daycare teachers and parents. The proactive connections between school learning and home practice may help strengthen the interactive influence between children and parents, promoting mutual understanding and reinforcing positive behaviors learned in educational settings. Strategies like private Facebook group discussions and virtual meetings with mindfulness activities for parents appear promising in overcoming barriers such as time constraints while fostering healthy communication and community support for sustained behavioral changes.

## Supplementary Material

jsaf010_Supplementary_Data

## Data Availability

Deidentified participant data can be requested from the PI.
