# Supplementary material for: Acceptability and satisfaction of a mindfulness-based healthy eating and stress management program targeting economically marginalized families in a pilot trial
Source: J Pediatr Psychol. 2025 Mar 17;50(5):402–11. doi: 10.1093/jpepsy/jsaf010 (PMC12112440; doi:10.1093/jpepsy/jsaf010)
Supplement: jsaf010_Supplementary_Data [file jsaf010_supplementary_data.docx]

**Teacher Evaluation Survey**

| Overall EVALUATION on the child CURRICULUM | | | | |
| --- | --- | --- | --- | --- |
|  | Strongly Disagree | Disagree | Agree | Strongly Agree |
| 1. The curriculum meets my expectations. | O | O | O | O |
| 1. The curriculum content is informative. | O | O | O | O |
| 1. The curriculum content is age appropriate for Head Start children. | O | O | O | O |
| 1. The curriculum instructions are easy to understand. | O | O | O | O |
| 1. I am confident that I can teach the lessons in the curriculum. | O | O | O | O |
| 1. The daily program length (20–30 minutes) is appropriate for Head Start children. | O | O | O | O |
| 1. The curriculum increases children’s knowledge on fruits and vegetables. | O | O | O | O |
| 1. The curriculum helps children eat more fruits and vegetables. | O | O | O | O |
| 1. I plan to continue teaching the curriculum in the future. | O | O | O | O |
| 1. Overall, I am satisfied with the curriculum. | O | O | O | O |
| 1. Children actively engaged in the following: |  |  |  |  |
| Healthy eating learning | O | O | O | O |
| Food taste-testing activity | O | O | O | O |

1. What changes, if any, would you suggest for the program?

___________________________________________________________________

___________________________________________________________________

___________________________________________________________________

1. What is the need for programs like *FirstStep2Health* in Head Start facilities?

___________________________________________________________________

___________________________________________________________________

___________________________________________________________________

1. What support or assistance do you need in order to independently teach the *FirstStep2Health* curriculum in the future?

**__________________________________________________________________**

**__________________________________________________________________**

**__________________________________________________________________**

**Parent Meeting Evaluation Surveys**

1. How satisfied are you with the yoga session?

- Not at all satisfied
- Not very satisfied
- Somewhat satisfied
- Very satisfied

1. How helpful are the contents covered at this meeting?

- Not at all helpful
- Not very helpful
- Somewhat helpful
- Very Helpful

1. Would you recommend this parent meeting to other parents?

Yes No

1. What is the biggest barrier that prevented you or other people you know from participating in the meeting?

_____________________________________________________________________

1. What else do you want to know in a parent meeting? How can we make the meeting better in the future?

­­­­­­­­­­­­­­­­­­­­­­­­­­­­­_______________________________________________________________________

_______________________________________________________________________

**Parent Program Evaluation Survey**

1. How often does your family use the cookbook provided? **(ONE answer ONLY)**

- Not at all (never used)
- Rarely (1 time a week)
- Sometimes (2 times a week)
- Often (3 or more times a week)

1. Would you have liked to receive something other than a cookbook to help you cook healthy foods?

Yes □ No

If “YES”, what would you have liked to receive to help you cook healthy foods?

___________________________________________________________________________

1. What is the average time you spent each week on the Facebook-based weekly tasks?

________________________minutes per week

1. The materials posted in the Facebook-based program were easy to understand.

- Disagree a lot
- Disagree a little
- Agree a little
- Agree a lot

1. The materials posted in the Facebook-based program assisted me in helping my family have a healthy lifestyle.

- Disagree a lot
- Disagree a little
- Agree a little
- Agree a lot

1. I will use the information that I learned from the Facebook-based program in the future.

- Disagree a lot
- Disagree a little
- Agree a little
- Agree a lot

1. Would you recommend the Facebook-based program to other parents you know?

Yes No

If “No”, please tell us why. __________________________________________________________________________________________________________________________________________________

1. What is the biggest barrier that stopped you from participating in the Facebook-based program?

_________________________________________________________________________

1. What else do you want to know in a Facebook-based program? How can we make it better in the future?

_________________________________________________________________________

_________________________________________________________________________

1. How helpful were the weekly letters made by your Head Start child?

- Not at all helpful
- Not very helpful
- Somewhat helpful
- Very Helpful

1. I talked about the weekly letters with my child.

- Disagree a lot
- Disagree a little
- Agree a little
- Agree a lot

1. I bought and prepared food for my child according to the child letters.

- Disagree a lot
- Disagree a little
- Agree a little
- Agree a lot

1. How can we make the child letters more helpful?

_________________________________________________________________________

_________________________________________________________________________

1. How satisfied are you with the whole program?

- Not at all satisfied
- Not very satisfied
- Somewhat satisfied
- Very satisfied

1. Would you participate in our program again if given chance?

- No, I do not think so
- Yes, I think so

**Caregiver Semi-Structured Interview Guide**

Thanks for taking the time to share your opinions with us. Keep in mind that we're just as interested in negative comments as positive comments, and at times the negative comments are the most helpful. We're tape recording the session because we don't want to miss any of your comments. Information collected from this session will be used by the researchers at Michigan State University to help them plan future programs. Information will only be presented in presentations or publications in group form. No individual names or identifiable information will ever be listed with a participant’s response.

**OPENING QUESTION:**

To start with, please tell me your overall experience participating in the program with your Head Start child.

PROBE: What are the activities you like most?

PROBE: What are the barriers/challenges that prevented you from participating in some activities?

**PARENT MEETINGS:**

Now let us talk about the parent meetings via zoom, please tell me what you like about the parent meetings.

What do you dislike about the parent meetings?

What about the meeting discussion contents? (e.g., program orientation, healthy cooking, and stress coping at Meeting 1; MyPlate, food labels, and smart shopping at Meeting 2; program review and community resources at Meeting 3)

What about the 30-minute yoga session at each meeting? What are your suggestions to improve it?

What do you think of the program cookbook? What other recipes do you want?

What are the barriers that prevented you from participating in the parent meetings?

What support do you need in order to attend the parent meetings in the future?

How can we make the parent meetings better together (format: face-to-face or virtual, location, discussion topic, duration, days of the week, time of the day)?

**FACEBOOK-BASED PROGRAM:**

Please tell me what you like about the Facebook-based program.

What do you dislike about the Facebook-based program?

What do you think about the weekly tasks and quiz (content, number, frequency)?

What are the barriers that prevented you from completing the weekly tasks or quiz?

How do others’ posting (type of post/content and frequency) influence your behaviors?

What else should we include in the Facebook-based program to help you create a healthy and happy family environment for your child?

How can we together make the Facebook-based program better?

**CHILD LETTER TO PARENTS:**

What do you think about your child’s letters?

How much has your child shared with you about the school program (e.g., the fruits and vegetables tried at Head Start)?

How does child’s participation (child letters) influence what you are doing at home related to healthy eating and cooking?

What can we do to make the child letters more effective?

Any comments on the school program for your child?

**ENDING QUESTION:**

How does the program in general influence your family’s thoughts and behaviors about healthy eating and life stress management?

Before we end our discussion, are there other thoughts, comments or suggestions that you would like to share with me to improve our children’s healthy eating behaviors and reduce parental stress, or to help us successfully involve parents in this type of research study?

**ENDING COMMENTS:**

Thank you very much for agreeing to participate in this discussion today. We really appreciate your time to share with us your thoughts and ideas. Your feedback will be most helpful as we develop a program to help our children become happier and healthier.
